# Insights into Transient Dimerisation of Carnitine/Acylcarnitine Carrier (SLC25A20) from Sarkosyl/PAGE, Cross-Linking Reagents, and Comparative Modelling Analysis

**DOI:** 10.3390/biom14091158

**Published:** 2024-09-14

**Authors:** Nicola Giangregorio, Annamaria Tonazzi, Ciro Leonardo Pierri, Cesare Indiveri

**Affiliations:** 1 CNR Institute of Biomembranes, Bioenergetics and Molecular Biotechnologies (IBIOM), Via Amendola 122/O, 70126 Bari, Italy; a.tonazzi@ibiom.cnr.it (A.T.); c.indiveri@ibiom.cnr.it (C.I.); 2 Department of Pharmacy—Pharmaceutical Sciences, University of Bari, Via E. Orabona, 4, 70125 Bari, Italy; 3 Department DiBEST (Biologia, Ecologia, Scienze della Terra) Unit of Biochemistry and Molecular Biotechnology, University of Calabria, Via Bucci 4C, 87036 Arcavacata di Rende, Italy

**Keywords:** mitochondrial carriers, oligomeric structure, homodimer, carnitine/acylcarnitine carrier (CAC), SLC25A20, purification–reconstitution–transport, cardiolipin, ping–pong kinetic mechanism, Sarkosyl-PAGE, heterobifunctional reagents, site-directed mutagenesis

## Abstract

The carnitine/acylcarnitine carrier (CAC) is a crucial protein for cellular energy metabolism, facilitating the exchange of acylcarnitines and free carnitine across the mitochondrial membrane, thereby enabling fatty acid β-oxidation and oxidative phosphorylation (OXPHOS). Although CAC has not been crystallised, structural insights are derived from the mitochondrial ADP/ATP carrier (AAC) structures in both cytosolic and matrix conformations. These structures underpin a single binding centre-gated pore mechanism, a common feature among mitochondrial carrier (MC) family members. The functional implications of this mechanism are well-supported, yet the structural organization of the CAC, particularly the formation of dimeric or oligomeric assemblies, remains contentious. Recent investigations employing biochemical techniques on purified and reconstituted CAC, alongside molecular modelling based on crystallographic AAC dimeric structures, suggest that CAC can indeed form dimers. Importantly, this dimerization does not alter the transport mechanism, a phenomenon observed in various other membrane transporters across different protein families. This observation aligns with the ping–pong kinetic model, where the dimeric form potentially facilitates efficient substrate translocation without necessitating mechanistic alterations. The presented findings thus contribute to a deeper understanding of CAC’s functional dynamics and its structural parallels with other MC family members.

## 1. Introduction

The carnitine/acylcarnitine carrier (CAC) is a member of the mitochondrial transporter family (SLC25), which facilitates the transport of various metabolites (including nucleotides, organic acids, coenzymes, and amino acids) across the inner mitochondrial membrane [1]. Among the 53 human mitochondrial carriers, CAC is a crucial component of the carnitine shuttle [2], enabling the translocation of fatty acids as acylcarnitines into the mitochondrial matrix for the β-oxidation pathway and ATP production [3]. The critical role of the CAC in energy metabolism is underscored by findings that mutations in its gene (SLC25A20) can cause a severe, early-onset disease (secondary carnitine deficiency) [4,5,6,7], characterised by profound cardiomyopathy, respiratory distress, and liver dysfunction [8,9].

Mitochondrial carriers primarily function as antiporters [10]; however, CAC also mediates the unidirectional transport of carnitine at a rate 1/10th of the antiport rate [11]. Notably, the kinetic mechanism of the CAC during substrate exchange is ping–pong (single binding site) [12], in contrast to other mitochondrial carriers proposed to facilitate substrate exchange via a sequential mechanism, where two binding sites are occupied simultaneously before the transport reaction occurs. Recent studies have shown that the ADP/ATP exchange, mediated by the ADP/ATP carrier (AAC) encoded by SLC25A4, also follows a ping–pong exchange mechanism [13]. It is believed that during substrate exchange, mitochondrial carriers (MCs) shift between two conformational states: the cytosolic state (c-state or c-conformation) and the matrix state (m-state or m-conformation) [14]. In the c-state, the carrier is open towards the intermembrane space and the substrate enters the carrier from the cytosolic side and binds to the similarly located substrate binding site shared by all the MCs [14]. As binding occurs, the carrier undergoes a structural rearrangement until it reaches a transition state, where optimal interactions between the carrier and substrate take place, following the ‘induced transition fit’ model of carrier catalysis [15]. In this transition state: (i) the substrate is positioned within the carrier’s central cavity, as suggested by the ‘single binding center-gating pore’ mechanism [16]; and (ii) the carrier is tightly structured around the substrate, with both sides of the membrane nearly sealed. The total binding energy of this optimal fit triggers further structural changes, resulting in the m-state, where the carrier is closed on the intermembrane space and open towards the mitochondrial matrix. At this point, the substrate, initially entered from the cytosolic side, is released into the matrix, allowing the cycle to continue with the entry of a counter substrate from the matrix [14]. This describes the MC-mediated antiport mechanism, though some carriers can also facilitate uniport, albeit at lower rates [14].

A longstanding debate regarding the functionality of carriers as monomers or oligomers has clarified that mitochondrial carriers (MCs) operate as monomers, with the substrate translocation pathway located within the cavity formed by six transmembrane helices [14,17,18]. However, proteins in the inner mitochondrial membrane can function as individual units or form supercomplexes to meet specific physiological demands (e.g., during fusion/fission events [19,20,21] or at contact sites with other organelles [22,23]). Thus, it is plausible that MCs may also form dimers/oligomers in response to specific physiological requirements (such as increased CAC units per mitochondrion due to the upregulation of lipid metabolism genes [2,24,25]) or structural necessities (like mitochondrial membrane curvature) [26,27,28,29,30], without altering their transport function. It is known that mitochondria from different tissues can form functional networks to meet tissue-specific demands [31]. Conversely, the dimerization/oligomerisation of mitochondrial carriers involved in forming large complexes (such as the ADP/ATP carrier in the permeability transition pore), or during fusion/fission events or contact sites with other organelles, might render them inactive as transport proteins. In such scenarios, protein packing is likely non-random and arranged along preferred interaction surfaces.

Remarkably, past research successfully reconstituted and studied the entire malate/aspartate shuttle, including the Aspartate/Glutamate Carrier (AGC) and the 2-oxoglutarate carrier (OGC) [32]. It has been suggested that AAC and phosphate carriers might form oligomers to participate in the permeability transition pore [33,34]. Recent findings propose that AGC forms a static unit through dimerization, facilitated by EF-hands within its calcium-binding motif [35]. In addition, it was recently proposed that intact mitochondrial membranes from *Bos taurus* yielded respiratory complexes and fatty acid-bound dimers of the ADP/ATP carrier [36]. The dimeric protein based on native mass spectrometry was again challenged by Kunji et al. [37] and Hirst et al. [38]. Hirst et al., claimed that the proteins ejected directly from mitochondrial membranes in the Chorev study were degraded, incorrectly assigned, and show discrepancies with ‘native states’ mostly obtained in detergent micelles. Kunji et al. instead claimed that discrepancies between the molecular weights attributed by Chorev et al., [36] were not due to post-translational modifications, according to molecular weights calculated by Kunji et al. [37], based on the electron density maps of the crystals. However, Chorev et al. demonstrated that all complexes are either ejected intact or in known intermediate states, with core subunit interactions maintained, without any degradation problem [39]. Conversely, it should also be stressed that Kunji et al. did not take into consideration that the ADP/ATP carrier, in the presence of a strong inhibitor, like carboxyatractyloside (Ki = 4 nM [40]), may undergo a different/reduced set of post-translational modifications, precisely due to the presence of the inhibitor, as observed for other proteins [41].

Here, our in vitro assays and in silico molecular modelling analyses provide new evidence regarding the assembly of MCs into homodimers/oligomers, and they suggest potential interaction surfaces. Specifically, we propose, through biochemical and molecular modelling approaches, that CAC, despite its ping–pong kinetic mechanism, may assemble as a homodimer, drawing parallels with the crystallographic dimers of AAC [42]. Our findings on CAC offer a novel interpretation of biochemical and biophysical data concerning the previously proposed homodimer formations of the phosphate carrier, citrate carrier, and uncoupling protein 2 [35,43,44,45,46,47].

## 2. Materials and Methods

### 2.1. Materials

PIPES, Triton X-100, Digitonin, egg yolk phospholipids (l- α phosphatidylcholine from fresh turkey egg yolk), cardiolipin, L-carnitine, N-Lauroylsarcosine sodium salt, Sulfo-MBS (m-maleimidobenzoyl-N-hydroxysulfosuccinimide ester), Sulfo-SMPB [Sulfosuccinimidyl 4-(p-maleimidophenyl)butyrate], N-ethylmaleimide (NEM), 1,4-Dithioerythritol (DTE), and Coomassie Brilliant Blue R-250 were purchased from Sigma-Aldrich, Milan, Italy; L-[methyl-^3^H]carnitine from Scopus Research BV Costerweg; Sephadex G-75, Sephadex G-200 from Pharmacia (Uppsala, Sweden); and anti-rabbit IgG horse radish peroxidase from Molecular Probes, Eugene, OR, USA, Clarity™ Western ECL Substrate from Bio-Rad, Hercules, CA, USA. All other reagents were of analytical grade.

### 2.2. Preparation of Rat Liver Mitoplasts and Purification and Reconstitution of Mitochondrial CAC in Proteoliposomes

Fresh rat liver mitochondria were purified using the standard procedure of cell disruption and differential centrifugation [48] before preparing mitoplasts by the use of digitonin [49]. Mitoplasts were extracted with 3% Triton X-100, and the CAC native protein was purified as described previously [50,51,52]. Furthermore, CAC from rat liver mitochondria, solubilised in Triton X-100 and partially purified on hydroxyapatite, was identified and completely purified through a specific elution on celite in the presence of cardiolipin. The complete purification of the CAC protein was confirmed through SDS gel electrophoresis, which revealed a single band in the purified celite fraction with an apparent molecular weight of 32,500 Da. In order to assess that the purified carrier was CAC, the different eluted fractions were reconstituted in a liposomal system by removing the detergent with a hydrophobic ion-exchange column (0.5 cm diameter, filled with 0.5 g of resin Amberlite), as previously described [51], for performing transport assays to verify that the reconstituted protein was able to catalyse the N-ethylmaleimide-sensitive carnitine/carnitine antiport typically observed by CAC [53]. In addition, the application of the above-reported protocol allowed us to identify the primary structure of the CAC starting from the protein purified from mitochondria, while no contaminant sequence was detected [52]. The concentration of intraliposomal carnitine was 15 mM in all samples.

### 2.3. Production, Purification, and Reconstitution of the CAC WT and C-lessV Mutant

The previously obtained cDNA of the CAC WT and C-lessV (a CAC mutant showing a valine residue in place of the native cysteine residues) mutant cloned in pMW7 [54] were used to overexpress and purify the CAC recombinant proteins [55]. A mixture composed of purified protein (60 μg), 1% Triton X-100, sonicated liposomes (10 mg of phospholipids), 10 mM PIPES pH 7.0, and 15 mM carnitine in a final volume of 680 μL was reconstituted as reported also for the protein purified from mitochondrial membranes (see above).

### 2.4. Transport Assays in Proteoliposomes

To test the transport activity of the CAC, 550 μL of proteoliposomes was passed through a *Sephadex G-75* column, in order to remove the external substrate. Six hundred μL of the turbid eluate was collected, divided into samples of 100 μL each, and used for transport measurement by the inhibitor-stop method [56]. The uptake was started by adding 0.1 mM [^3^H]carnitine to proteoliposome samples and at the indicated time interval stopped by 1.5 mM NEM (N-ethylmaleimide). In the control samples, NEM was added together with the labelled substrate at time zero. Transport rates were measured within 10 min, i.e., within the initial linear range of the time course, at 25 °C. After terminating the transport reaction, the external substrate was removed by chromatography on the *Sephadex G-75* column, and the intraliposomal radioactivity was measured [56]. The experimental values were corrected by subtracting the controls.

### 2.5. Sarkosyl/PAGE Analysis

The method employed for Sarkosyl/PAGE was similar to that of SDS/PAGE [50], except that the polyacrylamide slab gel electrophoresis (14% *w*/*v*) was performed in the presence of 0.05% *w*/*v* Sarkosyl, as reported by L. Huang et al. [57], totally replacing SDS (0.1% *w*/*v*), as well as the samples loaded on the gel, and the running buffer contained 0.05% Sarkosyl. Both Sarkosyl and SDS can be used to solubilise membrane proteins in their native state, but Sarkosyl is known for its milder solubilisation properties compared either to SDS [58,59], making it suitable for solubilising membrane proteins while maintaining their native structure [57,60]. Electrophoresis was performed at 100 Volt keeping the system at 4 °C. Notably, the size-exclusion chromatography of the CAC reconstituted into liposomes, i.e., after detergent removal (with reference to the Triton X-100 used in solubilisation) through hydrophobic chromatography (on Amberlite), we can estimate that the contribution of the detergent to the molecular weight estimation is not significant. In addition, the concentration of Sarkosyl (0.05%) used in this study to run the gel is below its CMC value (critical micelle concentration). Under these conditions, the stability of the investigated proteins is preserved [57,60], which is also in agreement with recent findings demonstrating that Sarkosyl can induce a helical structure typically observed for membrane proteins [57,60,61], allowing us to exclude problems either with the gel run or with molecular weight contributions.

### 2.6. Chemical Cross-Linking of Purified and Reconstituted CAC Protein

The chemical cross-linking of the purified and reconstituted mitochondrial carnitine/acylcarnitine carrier (0.5 µg protein) was performed by 0.5 mM Sulfo-MBS (m-maleimidobenzoyl-N-hydroxysulfosuccinimide ester) and/or Sulfo-SMPB [Sulfosuccinimidyl 4-(p-maleimidophenyl)butyrate]. After incubation for 30 min at room temperature, the cross-linkers were quenched by the addition of a buffer containing 10 mM DTE and 20 mM Tris-HCl, pH 8.0. Upon SDS-PAGE and immunostaining (see below), the mobility of the samples was determined.

### 2.7. Western Blotting

Proteins separated on 14% SDS-PAGE or Sarkosyl-PAGE were blotted onto the nitrocellulose membrane. The blot was blocked with 5% milk powder in Tris-buffered saline with 0.1% Tween 20 Detergent (TBST) for 30 min at room temperature (RT) with agitation. The membrane was then incubated with the self-produced polyclonal antibody against the carnitine/acylcarnitine carrier (dilution 1:2000) for 2 h at RT with agitation [62,63]. The primary antibody solution was washed 3 times for 15 min in TBST at RT with agitation. The blot was then incubated in a secondary antibody (anti-rabbit IgG horse radish peroxidase conjugated, from Molecular Probes) diluted to 1:20,000 for 1 h at RT with agitation. The blot was washed as above and developed for 5 min with Clarity™ Western ECL Substrate, according to the manufacturer’s instructions (Bio-Rad).

### 2.8. Gel Filtration Analysis

Thirty μg of the purified carnitine/acylcarnitine carrier was applied on a Sephadex G-200 gel filtration column (20 cm height and 1.5 cm diameter) equilibrated in buffer A (0.1% Triton X-100, 100 mM NaCl, 10 mM PIPES pH 7.0) and chromatographed at a flow rate of 0.3 mL/min with the same buffer. The detection of the CAC was monitored by the immunoblotting of the eluted samples. Five μg of Bovine Serum Albumin (BSA) and Carbonic Anhydrase (CA) were chromatographed on the same Sephadex G-200 gel filtration column as the molecular markers. The eluted fractions were detected by the Bradford dye-binding method (Bio-Rad protein assay).

### 2.9. 3D Molecular Modelling Analysis

The 3D comparative model of the CAC in c-conformation was built based on the structure of *Bos taurus* AAC1 (PDB_ID: 1okc) crystallised in complex with carboxyatractyloside, according to [18]. In addition, the 3D comparative model of the CAC in m-conformation was built based on the structure of the *Thermothelomyces thermophilus* AAC1 (PDB_ID: 6gci.pdb) crystallised in complex with bongkrekic acid and a nanobody, according to [18]. In order to propose a possible structure for the CAC dimer consisting of two monomers in c-conformation or in m-conformation or one monomer in c-conformation and one monomer in m-conformation, the structure of the yeast ADP/ATP carrier 2 (PDB_ID: 4c9h.pdb) [42] deposited as a crystallographic dimer, consisting of two parallel ADP/ATP carrier monomers in c-conformation, was used as a protein template for driving the preparation of 3D models of the CAC dimers.

Aiming to produce a 3D model of the CAC dimer consisting of two CAC monomers in c-conformation, which we shall call c-/c-CAC dimer, two monomers of the CAC 3D model in c-conformation (identified with chain X and chain Y) were superimposed to the two monomers of the ADP/ATP carrier 2 crystallographic dimer (4c9h.pdb, identified by chain A and chain B).

Then, two monomers of the CAC 3D model in m-conformation (identified by chain J and chain K) were superimposed to the two monomers of the ADP/ATP carrier 2 crystallographic dimer (4c9h.pdb, identified by chain A and chain B), for obtaining the 3D model of the CAC dimer consisting of two CAC monomers in m-conformation, which we shall call m-/m-CAC dimer.

In addition, a third 3D model of the CAC dimer hosting a CAC monomer in c-conformation (chain X) and another one in m-conformation (chain K), which we shall call c-/m-CAC dimer, was obtained by superimposing the CAC monomer in c-conformation (chain X) on the ADP/ATP carrier chain A of 4c9h.pdb, whereas the CAC monomer in m-conformation (chain K) was superimposed to the ADP/ATP carrier chain B of 4c9h.pdb.

Finally, a fourth 3D model of the CAC dimer hosting a CAC monomer in m-conformation (chain J) and another one in c-conformation (chain Y), which we shall call m-/c-CAC dimer, was obtained by superimposing the CAC monomer in m-conformation (chain J) on the ADP/ATP carrier chain A of 4c9h.pdb, whereas the CAC monomer in c-conformation (chain Y) was superimposed to the ADP/ATP carrier chain B of 4c9h.pdb.

Cardiolipin (CDL) unit coordinates surrounding the CAC 3D models in c-conformation were taken from 4c9h.pdb, whereas CDL unit coordinates surrounding the CAC 3D models in m-conformation were taken from the crystallised ADP/ATP carrier in m-conformation (6gci.pdb; [64]) along the superimposition operations.

The super command implemented in PyMOL 2.5.4 was used for guiding superimposition operations that allowed us to obtain all the above-described 3D models of possible CAC dimers, according to protocols described elsewhere [65,66,67].

### 2.10. Membrane Building and Energy Minimisation

After obtaining the above-cited CAC dimeric all-atom 3D models, we embedded the generated 3D models into a membrane bilayer consisting of POPC (phosphatidylcholine), POPE (phosphatidylethanolamine), and TMCL1 (cardiolipin), which are considered the main components of the mitochondrial inner membrane [68], by using the Membrane Builder plug-in and CHARMM topology available under the Charmm-GUI Effective Simulation Input Generator [69,70,71] to prepare the input files for a preliminary group of energy minimisation steps, in order to evaluate the stability of the generated 3D models and energetically minimise them.

The membrane bilayer was built up as a 115–118 Å side square POPC/POPE/TMCL1 patch for all the generated CAC dimer 3D models (see Table 1). In the setup phase, the psfgen tool has been used to generate a complete all-atom psf file of the system.

The obtained structure was solvated in a double layer of TIP3P (transferable intermolecular potential 3P) by setting the water thickness (minimum water height on the top and bottom of the system) to 25 Å.

Then, periodic boundary conditions with box dimensions 130 (x) × 130 (y) × 120 (z) Å and PME (particle mesh Ewald) were applied for each generated CAC dimer 3D model to compute full electrostatics for the generated systems.

The Include Ions plug-in was used to add K^+^ and Cl^−^ ions corresponding to a concentration of 200 mM to all the systems (according to previously validated protocols, [65,72]). The system was set to be neutralised with a protection shell of 5 Å from the protein, and a minimum distance of 5 Å between ions was imposed. For an idea of the system size, see Table 1.

The simulation temperature was kept constant at 300 K by means of a Langevin thermostat, and the pressure was set to 1 atm (101.325 kPa) using a Langevin piston during the minimisation and equilibration protocol for which an integrator time step was set to 2 fs and the ‘rigidbonds’ parameter was set to ‘all’. Starting from the above-described systems, 50,000 equilibration (conjugate gradient) steps were performed. All the equilibration steps were performed by using NAMD2 with the force field CHARMM36m with cmap correction [69,70,71], according to protocols previously described [65,72].

**Table 1:** System composition of the simulated dimeric complexes.

	c-/c-CAC Dimer	m-/m-CAC Dimer	c-/m-CAC Dimer	m-/c-CAC Dimer
Number of Atoms	148,922	139,584	146,885	145,207
Crystal type	tetragonal	tetragonal	tetragonal	tetragonal
System size (Å)	(A) 117.75 × (B) 117.75 × (C) 116.15	(A) 115.91 × (B) 115.91 × (C) 112.47	(A) 115.81 × (B) 115.81 × (C) 118.05	(A) 115.73 × (B) 115.73 × (C) 117.16
Crystal angle (degrees)	(alpha) 90 × (beta) 90 × (gamma) 90	(alpha) 90 × (beta) 90 × (gamma) 90	(alpha) 90 × (beta) 90 × (gamma) 90	(alpha) 90 × (beta) 90 × (gamma) 90
Number of lipids	300 (158 POPC; 98 POPE; 44 CL)	290 (153 POPC; 95 POPE; 42 CL)	290 (153 POPC; 95 POPE; 42 CL)	290 (153 POPC; 95 POPE; 42 CL)
Number of water molecules (TIP3P)	31,988	29,307	31,766	31,208
Number of K^+^ ions	108	92	102	101
Number of Cl^−^ ions	82	75	82	81

### 2.11. Interaction Energy

The 3D coordinates of the dimeric CAC 3D models were used to estimate the binding affinity between CAC monomers at the CAC-CAC protein–protein interface by using the FoldX AnalyseComplex assay [65,66,73,74]. Indeed, the FoldX AnalyseComplex assay was performed to determine the interaction energy between the investigated minimised protein complexes consisting of the CAC/CAC dimer. The way the FoldX AnalyseComplex operates is by unfolding the selected targets and determining the stability of the remaining molecules and then subtracting the sum of the individual energies from the global energy [65,66,73,74]. More negative energy values indicate a better binding, whereas positive energy values indicate no binding [65,66,73,74].

### 2.12. Other Methods

The measurement of the total protein concentration in the mitoplast extract was based on the Bradford dye-binding method (Bio-Rad protein assay). The protein amount was also estimated from Sarkosyl-PAGE or SDS-PAGE gels stained with Coomassie Brilliant Blue R-250 by using the Chemidoc imaging system equipped with Quantity One 4.5 software (Bio-Rad, Hercules, CA, USA), as previously described [54].

### 2.13. Statistical Analysis

A statistical analysis was performed using Student’s *t*-test, as indicated in the figure legends. Values of * *p* < 0.01 were considered statistically significant. Data points were derived from the means of three different experiments, as specified in the figure legends.

## 3. Results

### 3.1. Dimer Detection of the Purified Carnitine/Acylcarnitine Carrier (CAC) by Sarkosyl/PAGE

**Figure 1:**
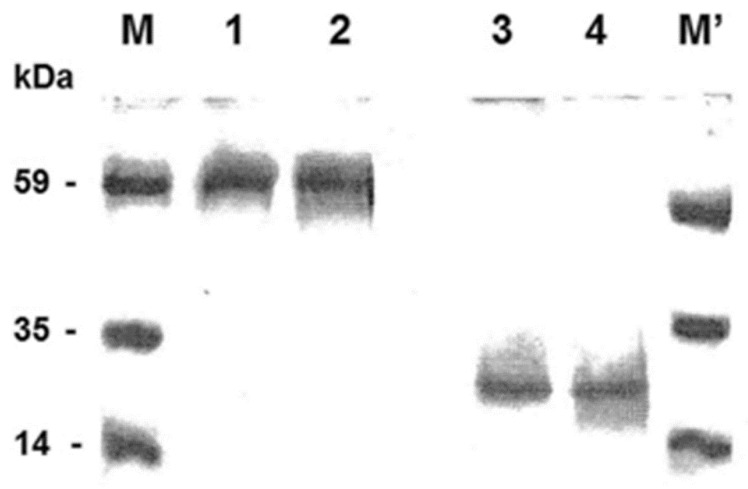
Sarkosyl-PAGE of the carnitine/acylcarnitine carrier isolated and purified from rat liver mitochondria detected by silver nitrate staining. Lanes 1 and 3 show purified CAC in Triton X-100, and lanes 2 and 4 show purified CAC incorporated into liposomes. Marker (M): lanes 1 and 2 were solubilised in 0.05% *w*/*v* Sarkosyl. M’: lanes 3 and 4 were pre-treated with 0.1% *w*/*v* SDS. Molecular weight standard markers: Protein Kinase B (PK B), Malic dehydrogenase (MD), and Phospholipase A2 (PLA2).

In order to determine the native oligomeric state of the CAC, a Sarkosyl/PAGE was performed according to L. Huang et al. [57,60] (see Section 2), which recently demonstrated that the anionic detergent Sarkosyl 0.05% *w*/*v* can preserve the native (biologically active and correctly folded) conformation of proteins, while allowing their masses to be identified by gel electrophoresis or mass spectrometry. As shown in Figure 1, the native carnitine/acylcarnitine carrier purified from isolated rat liver mitochondria [51,52] exhibits an apparent electrophoresis mobility corresponding to a molecular weight of 59 kDa (Figure 1 lane 1), i.e., about twice that of the protein monomer [50,75]. On the contrary, the protein solubilised with a sample buffer containing 0.1% SDS, before running on the same mild non-denaturing gel, displayed an apparent molecular weight of 25.5 kDa (Figure 1 lane 3), which is slightly lower than the apparent molecular weight observed on SDS-PAGE [50,75]. This irregular behaviour during gel separation may be due to the different migration properties of the CAC compared to the soluble proteins used as markers [76]. An apparent molecular weight of 59.0 kDa and 25.5 kDa was also found after protein extraction from proteoliposomes by 0.05% Sarkosyl and 0.1% SDS, respectively (Figure 1 lanes 2 and 4). As the control, the transport activity ([^3^H]-carnitine/carnitine homo-exchange) of the reconstituted protein was measured. It was 2.5 μmol/10 min/g protein, which is coherent with previous estimations [50].

### 3.2. The Detection of the Dimeric Form of the Purified and Reconstituted CAC Protein Following Western Blotting

**Figure 2:**
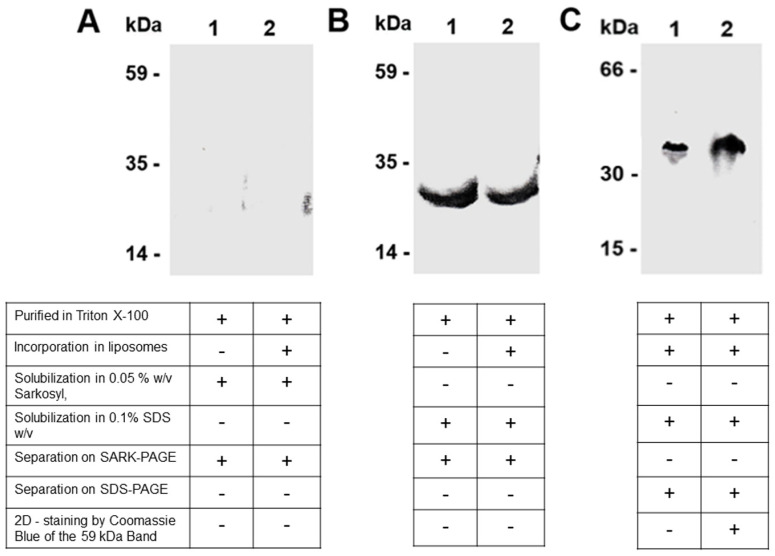
An immunoblot of the carnitine/acylcarnitine carrier separated on Sarkosyl-PAGE. (**A**) Purified CAC in Triton X-100 (lane 1) and purified CAC incorporated into liposomes (lane 2) were solubilised in 0.05% *w*/*v* Sarkosyl, separated on Sarkosyl-PAGE, as described in ‘Section 2’, and immunostained by an anti-CAC antibody. (**B**) Purified CAC in Triton X-100 (lane 1) and purified CAC incorporated into liposomes (lane 2) were solubilised in 0.1% *w*/*v* SDS, separated on Sarkosyl-PAGE, and immunostained by an anti-CAC antibody. (**C**) The band of the reconstituted CAC protein separated on Sarkosyl-PAGE at 59 kDa (as highlighted in Figure 1, lane 1 and lane 2) and stained by Coomassie Blue (see Figure 1) was excised and applied to a denaturing second dimension (2D) SDS-PAGE (lane 2), together with the reconstituted purified carnitine carrier as the control (lane 1). Molecular weight standard markers: Protein Kinase B (PK B), Malic dehydrogenase (MD), Phospholipase A2 (PL A2) for Sarkosyl-PAGE and Bovine Serum Albumin (BSA), and Carbonic Anhydrase (CA) and Cytochrome C (Cyt C) for SDS-PAGE. See the Supplementary Materials for uncropped blots.

The dimeric composition of the CAC, after the separation of the protein on a non-denaturing gel, was also detected by Western blotting. Indeed, the purified and reconstituted protein separated on a SARK-PAGE (0.05% Sarkosyl) was subjected to immunostaining with a specific polyclonal antibody against the whole CAC (see Section 2 and [62,63]). No immunodetection was distinguished both in the purified and reconstituted protein (Figure 2A), whereas immunodecoration became visible at the apparent molecular weight of 25.5 kDa, when a duplicate of the same protein sample was solubilised by SDS (Figure 2B). To demonstrate that all detergent molecules were removed by Amberlite during the reconstitution procedure (see Section 2), and thus do not affect the immunodetection of proteoliposomes, we performed the Chadda protocol [77] with some variations. It consisted of subjecting tubes containing proteoliposomes to four freeze–thaw cycles (dry ice/ethanol bath alternating with water bath for thawing). At the end of the procedure, the proteoliposomes were turbid and precipitated to the bottom of the tubes, indicating that no detergent molecule was present. These data indicate that the major antigenic portion of the CAC structure, in our gel in non-denaturant conditions, is not accessible when the carrier is both in solution and folded into proteoliposomes. However, to evaluate the existence of the dimeric form of the protein, which did not react with the antiserum, other decisive experiments were performed. In the first step, we ran the reconstituted CAC protein on the non-denaturing preparative gel in the presence of 0.05% Sarkosyl; afterwards, the highlighted band with an apparent molecular weight of 59 kDa (as shown in Figure 1), stained by Coomassie Blue G-250 (BIO-RAD), was excised and applied to a denaturing SDS-PAGE system (containing 0.1% SDS) (Figure 2C lane 2) together with the reconstituted purified carnitine carrier as the control (Figure 2C lane 1), prior to Western blotting. Both samples, under denaturing conditions, were immunodetected at 25.5 kDa, indicating that the 59 kDa band excised from the gel corresponded to the dimeric form of the carnitine/acylcarnitine carrier.

### 3.3. The Detection of the Dimeric Form of the Purified and Reconstituted CAC Protein by the Chemical Cross-Linking Strategy

**Figure 3:**
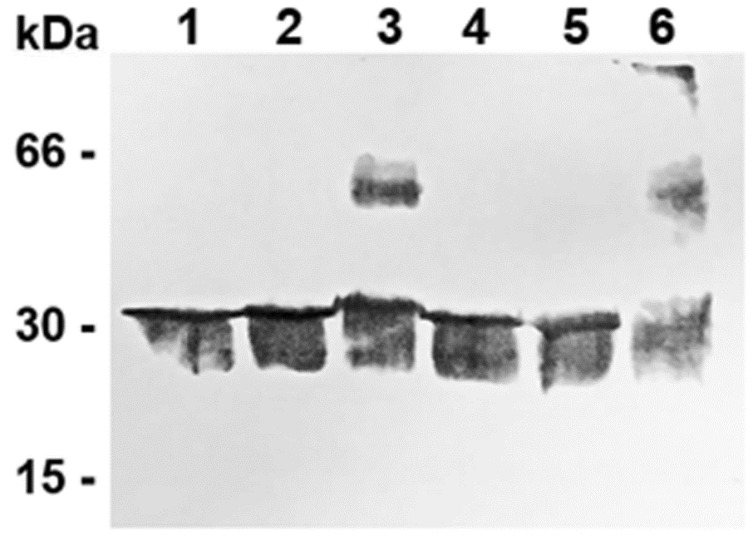
The immunodetection of the dimeric/oligomeric structure of the reconstituted mitochondrial CAC protein using chemical cross-linking reagents. The reconstituted native CAC protein was pre-treated with Sulfo-MBS and Sulfo-SMPB, see ‘Section 2’ for details, before the separation of the samples on SDS-PAGE and Western blotting. Untreated samples (lanes 1 and 4); samples treated with Sulfo-MBS and Sulfo-SMPB (lanes 3 and 6); and negative controls (lanes 2 and 5) in which the samples were quenched by the addition of a buffer containing 10 mM DTE and 20 mM Tris-HCl, pH 8.0 before treatment with Sulfo-MBS and Sulfo-SMPB. Molecular weight markers: Bovine Serum Albumin (BSA), Carbonic Anhydrase (CA), and Cytochrome C (Cyt C). See the Supplementary Materials for uncropped blots.

The quaternary structure of the CAC was also demonstrated by a chemical cross-linking strategy. This approach has been successfully used for different transporters [78] and consists of using cross-linking reagents to covalently link the neighbouring monomers of the protein. Previously, we treated rat liver mitoplasts with formaldehyde, whose cross-linking distance is 2.3–2.7 Å, to demonstrate the binding between CPT-2, anchored to the inner mitochondrial membrane, facing the matrix, and CAC embedded in the inner mitochondrial membrane, and thus the formation of a supramolecular complex whose function is to channel acylcarnitines from CAC to CPT-2 and allow the ß-oxidation of fatty acids occurred [62]. In contrast, in the case of the purified CAC protein, formaldehyde was ineffective. Although it results in efficient cross-linking, formaldehyde is small and is known to react non-specifically with different functional groups of membrane proteins, showing limitations in the protein structure determination. Likely, in the case of the CAC, the relative higher mobility of the protein and the greater distance between two neighbouring CAC monomers rendered ineffective the employment of formaldehyde to reveal/induce stable CAC dimers, in the previously described conditions. In contrast, as widely reported in the literature, it is more convenient to investigate the membrane protein quaternary structure using heterobifunctional cross-linkers, which specifically react with Lys and Cys residues and stabilise membrane protein complexes [79]. In order to ascertain that the two monomers can be linked by a longer cross-linker, we found that Sulfo-MBS [m-maleimidobenzoyl-N-hydroxysulfosuccinimide ester], M.W. 416.30, spacer arm length 9.9 Å and Sulfo-SMPB [Sulfosuccinimidyl 4-(p-maleimidophenyl)butyrate], M.W. 458.38, and spacer arm length 14.5 Å were effective for cross-linking monomers of the protein. These water-soluble heterobifunctional reagents contain NHS-ester (which can bind amine-containing molecules, i.e., lysine and arginine residues) and maleimide (which can bind sulphydryl-containing molecules, i.e., cysteine residues) reactive groups at opposite ends. We tested these cross-linkers on the reconstituted protein. The samples were treated with 0.5 mM Sulfo-MBS or Sulfo-SMPB for 30 min (see Section 2 for details), separated by SDS-PAGE, i.e., under denaturing conditions, and were immunostained with a CAC polyclonal antibody. Sulfo-MBS and Sulfo-SMPB-treated samples clearly showed an apparent molecular weight double (Figure 3, lanes 3 and 6), compared to the untreated reconstituted purified protein (Figure 3, lanes 1 and 4), indicating the structural association of the two monomers. Remarkably, a limitation of using chemical cross-linking is the high risk of detecting non-specific interactions by running the optimised protocol [80]. In fact, to avoid the formation of undesired subunit interactions, we were forced to stop the incubation of the reconstituted protein with cross-linkers after 30 min (see Section 2), which was an insufficient amount of time to observe a complete dimerization reaction. However, the Sulfo-MBS cross-linking reaction was not complete; indeed, a considerable amount of protein still migrated as a monomer. As a negative control, the addition of 10 mM DTE and 20 mM Tris-HCl, pH 8.0 before the reaction of proteoliposomes with cross-linkers prevented dimer formation (Figure 3, lanes 2 and 5). The immunodecoration following the treatment of the reconstituted protein with Sulfo-SMPB also displayed the existence of an apparent band at about 90 kDa (Figure 3, lane 6).

### 3.4. Different Ratios of Recombinant WT and C-lessV Proteins Reconstituted in Liposomes Lead to Changes in the Dimeric Structure of the CAC

**Figure 4:**
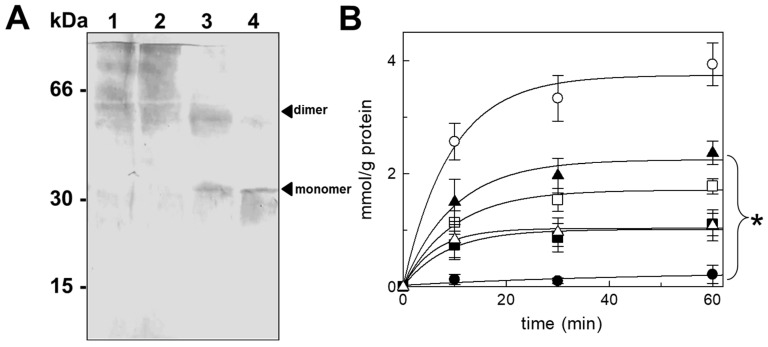
The immunodetection of the oligomeric structure of reconstituted CAC recombinant proteins (WT and C-lessV) by a chemical cross-linking reagent. (**A**) Different ratios of recombinant CAC reconstituted WT/C-lessV proteins (100 lane 1, 75/25 lane 2, 50/50 lane 3, and 25/75 lane 4) incubated with Sulfo-SMPB, see ‘Section 2’ for details, before SDS-PAGE and Western blotting (for lane 1, see the SEC chromatogram of Figure S1). Molecular weight markers: Bovine Serum Albumin (BSA, 66 kDa), Carbonic Anhydrase (CA, 30 kDa), and Cytochrome C (Cyt C, 15 kDa). (**B**) The time course of WT and C-lessV reconstituted separately or mixed in combination (50/50 ratio): WT homodimer (○), WT homodimer + NEM (●), C-lessV homodimer (□), C-lessV 50% homodimer (■), WT/C-lessV (▲), and WT/C-lessV + NEM (△). The values are the means ± SD from three independent experiments. The * indicates that the activity of each tested combination is significantly different (lower) from the respective control (WT homodimer), as estimated by Student’s *t*-test (* *p* < 0.01). See the Supplementary Material for uncropped blots.

Interestingly, there was evidence that the oligomeric structure of the CAC decreases in cross-linking with sulfo-SMPB when different ratios of recombinant proteins, i.e., WT and the active Cys residue-free mutant (C-lessV), were added to the reconstitution mixture prior to SDS-PAGE and immunoblotting (Figure 4A). Indeed, the presence of an increasing amount of recombinant cysteine-free protein led to a progressive stochastic formation of the WT homodimer, which disappeared completely at the ratio 25/75 WT/C-lessV. In lanes 1 and 2, smears due to the possible random aggregation of the recombinant proteins in the presence of the hetero-cross-linking agent are evident at the higher molecular weights. Similar smears are totally absent in purified mitochondrial CAC reconstituted in liposomes before treatmeant with sulfo-SMPB (Figure 3). A duplicate of the sample loaded onto the gel with the 50/50 ratio WT/C-lessV, not treated with sulfo-SMPB, was used to determine the transport activity of the CAC reconstituted in the liposomes. The incorporation of the C-lessV mutant into liposomes was similar to that of WT, as we previously demonstrated for several CAC recombinant proteins [54]. The time course in Figure 4B shows the variation in transport activity as a function of the 50/50 ratio between WT and C-lessV (▲), compared to recombinant proteins alone (only WT, ○; only C-lessV, □), which led us to assume that CAC may be in a dimeric active form in the lipid membrane. However, we also highlighted that while a complete inhibition of the CAC transport activity was observed in the sole WT treated with 30 μM N-ethyl-maleimide (NEM) (●), the reconstituted mixture of WT/C-lessV (50/50 ratio) incubated with NEM exhibited a partial functionality (△), most likely due to no inhibitor reactivity towards C-lessV. To confirm this hypothesis, we tested the transport activity of the C-lessV alone added to the reconstitution mixture in the same amount as the WT/C-lessV, i.e., 50%. Since the transport activity of C-lessV with that amount of protein (■) was similar to that of the WT/C-lessV hetero-dimer (50/50 ratio) incubated with NEM (△), we can deduce that in the latter case the protein composition of the proteoliposomes was a mixture of both recombinant proteins. Therefore, these data indicate that CAC may assemble as a dimer (Figure 4A), but can also function as a monomer when assembled as a dimeric structure.

### 3.5. The Identification of the Dimeric Form of Purified CAC Protein by Size-Exclusion Chromatography

The molecular mass of the native CAC protein purified from rat liver mitoplasts was analyzed also by size-exclusion chromatography (Sephadex G-200). With some appropriate modifications, a similar procedure was used for the mitochondrial phosphate [43] or citrate carriers [44]. As shown in Supplementary Figure S1A, the SDS-PAGE profile of the purified CAC was coincident with that of BSA (Bovine Serum Albumin = 66 kDA), with a peak at fraction 6, whereas CA (Carbonic Anhydrase = 30 kDa) elution is delayed (fraction 9), which is consistent with the smaller expected monomer size. Finally, the elution profile of the carrier was confirmed by Western blotting (Supplementary Figure S1B). Thus, the molecular masses of the native CAC and BSA were similar. Sephadex protein eluate (300 μL) was also reconstituted into liposomes to detect the transport activity. The trend in transport activity followed the elution curve (Supplementary Figure S1C).

### 3.6. 3D Molecular Modelling and Energy Minimisation

**Figure 5:**
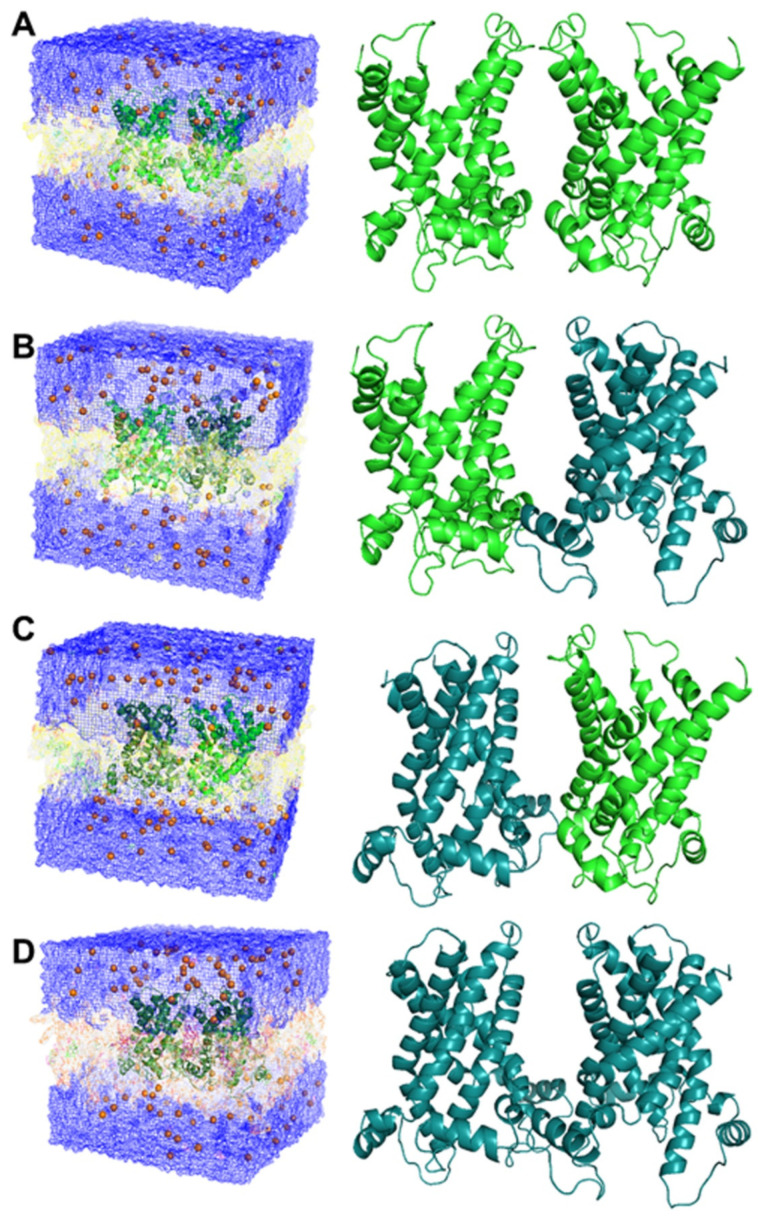
3D models of the CAC dimer consisting of two monomers in c-conformation and/or in m-conformation. Panel (**A**): the 3D model of the CAC dimer consisting of two monomers in c-conformation is reported in the light green cartoon representation. Panel (**B**): the 3D model of the CAC dimer consisting of two monomers, c-conformation (light green) on the left, and m-conformation (dark green) on the right, is reported in the cartoon representation. Panel (**C**): the 3D model of the CAC dimer consisting of two monomers, m-conformation (dark green) on the left and c-conformation (light green) on the right, is reported in the cartoon representation. Panel (**D**): the 3D model of the CAC dimer consisting of two monomers in m-conformation is reported in the dark green cartoon representation. All the reported CAC dimers are embedded in a lipid bilayer consisting of POPC, POPE, and CDL (orange mesh representation). Water molecules are reported in the blue mesh representation, and K^+^, Cl^−^ ions are reported in the light/dark-orange spheres. The number of atoms and molecules present in the system is indicated in the related Section 2. The four panels on the right highlight the only protein dimer portion. The reported dimers built from the last frames obtained through the energy minimisation procedures (50,000 steps of energy minimisation) were then used for calculating the free energy of the interaction (see the following paragraph).

In order to have an idea of the possible CAC dimeric overall structure, the obtained 3D models of the CAC in c- and m-conformation [18] were alternatively superimposed to the crystallographic dimer of the ADP/ATP carrier (4c9h.pdb) to obtain two adjacent monomers of the CAC in c-conformation (c-/c-CAC dimer) or in m-conformation (m-/m-CAC dimer), and two other protein dimers (c-/m-CAC dimer and m-/c-CAC dimer), hosting a CAC monomer in c-conformation and a second CAC monomer in m-conformation (Figure 5).

After completing the energy minimisation protocol, as described in the Section 2, interactions occurring at the protein–protein interface were checked in all the relaxed models. First of all, a group of 24 residues forming reciprocal H-bonds and ion pairs is observed (minimum distance below 4 Å) in the bottom half of the CAC dimer hosting two CAC monomers in c-conformation, between the short helix h12 (one of the three helices parallel to the membrane plane, [81]) of both monomers and the residues of the matrix loop mlb56 (located between the short helix h56 and transmembrane helix H6, at the level of the second part of mitochondrial carrier sequence motif, [81]) (Table 2).

In the upper half of the CAC dimer hosting two CAC monomers in c-conformation, we can observe a set of reciprocal H-bonds and ion pairs between the residues located between the end of the transmembrane helix H4, residues of the cytosolic loop h45, and residues of the transmembrane helix H5 (for a description about the length of the CAC and other MC family members, see Pierri et al. [81]). Some weaker hydrophobic interactions are observed between the residues on the transmembrane helix H5 of the two monomers, located between the two PG-levels [18,81] (Table 2).

Then, a group of 37 interacting residues forming reciprocal H-bonds and ion pairs is observed (minimum distance below 4 Å) in the bottom half of the CAC dimer, hosting two CAC monomers in m-conformation, between the short helix h12 (one of the three helices parallel to the membrane plane, [81]) of both monomers and the matrix loop mlb56 (located between the short helix h56 parallel to the membrane plane and transmembrane H6, which is at the level of the second part of mitochondrial carrier sequence motif. For a description about the length of the CAC secondary structure elements and other features of MC family members, see Pierri et al. [81]). In the upper half of the CAC dimer, hosting two CAC monomers in m-conformation, we can observe a set of reciprocal H-bonds and ion pairs between the C-terminal portion of the transmembrane helix H4, the cytosolic loop h45, and the N-terminal portion of the transmembrane helix H5 [81]. An additional set of interactions is observed at the middle region of the CAC dimer, and in the region defined by the two PG-levels [18,81] between transmembrane helix H5 and transmembrane helix H6 (for a description about the length of the CAC and other MC family members, see Pierri et al. [81]) (Table 1).

Finally, a group of 29 interacting residues forming reciprocal H-bonds and ion pairs is also observed (minimum distance below 4 Å) in the bottom half of the other two CAC dimer 3D models, hosting a CAC monomer in c-conformation and the other one in m-conformation, between the short helix h12 of both monomers and the matrix loop mlb56 (located between the short helix h56 parallel to the membrane plane and transmembrane helix H6, at the level of the second part of mitochondrial carrier sequence motif). In the upper half of the same CAC dimer 3D model, we can observe a set of reciprocal H-bonds and ion pairs between the C-terminal portion of the transmembrane helix H4, the cytosolic loop h45, and the N-terminal portion of transmembrane helix H5. An additional set of interactions is observed at the middle level of the CAC dimer between the PG-levels [18,81], involving residues located on transmembrane helix H5 and transmembrane helix H6 (Table 2).

**Table 2:** Residues at the monomer–monomer interface forming interactions at the protein regions are indicated in the first column between the secondary structure elements indicated in the second column. The first letter of the reported residues indicates the amino acids in the 4 Å distance range, whereas the second letter indicates the specific protein chain within the investigated dimer.

		c-conf/c-conf CAC Dimer		m-conf/m-conf CAC Dimer		c-conf/m-conf CAC Dimer		m-conf/c-conf CAC Dimer	
Protein Dimer Regions	Secondary Structure Elements	Residues at the Protein–Protein Interface		Residues at the Protein–Protein Interface		Residues at the Protein–Protein Interface		Residues at the Protein–Protein Interface	
Residues involved in interactions in the CAC dimer bottom half (between matrix gate area and matrix loops)			QY41	SJ53	SK53		SK53	SJ53	LY38
	h12	SX53	SY53	TJ55				RJ60	PY42
		IX56	IY56	IJ56	IK56	IX56	IK56		SY53
				DJ57	DK57	DX57	DK57		TY55
		RX60	RY60	RJ60	RK60		RK60		IY56
				KJ61	KK61		KK61		
								
								IJ257	
								DJ259	
			EY260	EJ260	EK260	EX260		EJ260	EY260
	h56-ml56b			VJ262	VK262	VX262		VJ262	
		TX263	TY263	TJ263	TK263	TX263		TJ263	TY263
			SY264	YJ266	YK266				
			KY267	KJ267	KK267		KK267		
								
									
Residues involved in interactions in the CAC dimer upper half (between the cytosolic gate area and cytosolic loops)	H4–H5	LX193							
		IX196						FJ197	
		FX197	FY197		FK197	FX197	FK197		
		TX198	TY198		TK198	TX198	TK198	TJ198	TY198
		PX199		EJ200	EK200	PX199		EJ200	VY204
			LY207	VJ204	VK204	LX207			
				PJ210			PK210		PY210
		RX211	RY211	RJ211		RX211	RK211	RJ211	RY211
					LK213		LK213		
				VJ214	VK214	VX214	VK214	VJ214	VY214
				AJ215		AX215			AY215
									
Residues involved in interactions in the CAC dimer middle regions (between PG-levels)	H5	FX218	FY218	FJ218	FK218	FX218	FK218	FJ218	FY218
	H6				NK270	NX270			
					FK277	IX274	NK270	NJ270	NY270

### 3.7. Interaction Energies

To investigate more deeply the stability of interactions occurring between the two monomers in the four complexes, we estimated the interaction energy at the protein–protein interface in the generated 3D models hosting the cited CAC dimers. It was observed that the CAC dimer hosting two monomers in m-conformation shows a slightly lower free energy of interaction (namely, a slightly more stable complex) than the one calculated for the CAC dimer hosting two monomers in c-conformation (Table 3). Notably, the lowest free energy of interaction (highest stability) is observed in one of the CAC dimers hosting a monomer in c-conformation and a monomer in m-conformation (Table 3).

## 4. Discussion

In this paper, through biochemical approaches and an in silico molecular modelling analysis, the quaternary structure of the mitochondrial carnitine/acylcarnitine carrier (CAC) has been investigated. Previously, we demonstrated that CAC establishes disulfide bridges either by chemical (Cu^2+^-phenanthroline oxidation) or spontaneous (molecular O_2_) oxidation of close cysteine residues within a single polypeptide chain [50]. In this case, the dimerization/oligomerisation of the CAC was studied on the purified mitochondrial protein for studying the protein in a more native-like environment. The CAC protein ability in forming the dimer/oligomer was investigated by chemical approaches using non-denaturing gel (SARK-PAGE), heterobifunctional cross-linkers (sulfo-MBS and sulfo-SMPB), and size-exclusion chromatography.

### 4.1. The Choice of Sarkosyl in the Solubilisation and Separation of CAC Dimers

We decided to use Sarkosyl (0.05%)-PAGE instead of SDS-PAGE or BN-PAGE because Sarkosyl is often considered milder than SDS, making it more compatible with maintaining native protein structures, compared to SDS [58,59] when dealing with integral membrane proteins (like our mitochondrial transporters) solubilisation [57,60]. While it is expected that BN-PAGE can slightly improve oligomer separation, the harsher properties of the dye interacting with membrane proteins let us prefer Sarkosyl in our strategy aiming to preserve, as much as possible, a folded state for the single monomers of our CAC. Indeed, it is believed the formation of oligomers and supercomplexes in the inner mitochondrial membrane strongly depend on the correct folding of the single monomers [28,83,84,85,86]. Notably, mitochondrial transporters are integral membrane proteins, with cytosolic and matrix loop playing a crucial role in membrane protein insertion and transport function [81]. Thus, we assumed that the employment of Sarkosyl (0.05%)-PAGE would have affected the folding of CAC units also at the level of the more accessible cited loops less, which are used for preserving their structure along the solubilisation and separation steps [57,60,87].

It should be noticed that, in the main papers on the topic from the Kunji group, the dimers of the mitochondrial carrier were not observed, most likely due to the employment of harsher detergents (alkyl(C8-13)-maltoside, LAPAO, digitonin, Triton X-100, C12E8, SDS, and cyclohexylmaltoside (CYMAL (C4-C7)) detergents) [17,88,89,90]. Notably, Sarkosyl was apparently never used in their protocols for investigating mitochondrial carrier dimerization [17,88,89,90]. Conversely, in more recent papers from the same group, Kunji et al. showed that AGC can form dimers. It was proposed that AGC dimerization was mediated by the AGC N-terminal portion, and dimer formation did not alter the AGC transport mechanism [35]. However, it was also shown in the past that AGC deprived of the N-terminal domain, reconstituted in proteoliposomes, showed an activity comparable to the activity of the full-length AGC ([91] and references therein), which led us to hypothesise that mitochondrial carrier dimer formation might also be ascribed to intermonomer helix–helix interactions.

Remarkably, it was recently proposed that intact mitochondrial membranes from *Bos taurus* yielded respiratory complexes and fatty acid-bound dimers of the ADP/ATP carrier by mass spectrometry [36], re-opening the debate about the ability of mitochondrial carriers to fold as dimers [36,39].

In light of all the above-reported observations and our findings, we believe that in our analysis it was possible to observe CAC dimers thanks to the employment of Sarkosyl (0.05%)-PAGE, which allowed us to observe CAC dimerization with both purified protein in solution and purified protein reconstituted in liposomes. Our data, following the gel electrophoresis of the purified protein under mild denaturation conditions [57,60], as demonstrated for other transporters [92], let us suppose that CAC can really assemble as a homodimer (Figure 1, lanes 1,2). Conversely, we also observed that SDS treatment (Figure 1, lanes 3,4) disrupts every interaction between the two monomers.

### 4.2. Detection of the Solubilised Purified/Recombinant CAC by Western Blot

When the purified or the reconstituted CAC were separated on the non-denaturing Sarkosyl gel and subjected to Western blotting, it was not possible to observe the quaternary structure at 59 kDa by a specific polyclonal antibody against the whole mitochondrial carnitine/acylcarnitine carrier. The missed detection of the dimer at 59 kDa was ascribed to the inaccessibility of the epitopes responsible for the antibody recognition, being involved in inter-monomer interactions. In support of this hypothesis, it was indeed observed that in the presence of denaturing SDS ionic detergent, the same antibody can reach the epitopes of the protein (as observed in Figure 2B). As a further confirmation, interestingly, when the band excised in Blue Coomassie at 59 kDa was loaded onto a denaturing gel (SDS/PAGE), the CAC protein was immunodetected as a monomer (Figure 2C). Our data fit with several cases indicating that membrane proteins show a quaternary structure in the detergent-solubilised state and exist in solution at equilibrium as different polypeptide species [93,94]. Furthermore, the formation of micelles between intrinsic proteins and a non-ionic detergenst, such as Triton X-100, which is also the detergent we used during the complete purification of the CAC from rat liver mitochondria, does not prevent the dimer from being formed [79,95].

### 4.3. Possible Role of Cardiolipin in Dimerisation along the Solubilisation/Separation Steps

Likely, in the case of the CAC, the curved surface of micelles does not affect both the tertiary structure and the oligomeric state, since, as observed for the crystallographic dimers of the ADP/ATP carrier [96,97], the protein-bound endogenous cardiolipin molecules can mimic the amphipathic mitochondrial membrane [98]. Since the ADP/ATP carriers, but also CAC, need cardiolipin to improve or preserve their functionality [99,100,101,102], the stability of the CAC dimer may be due to the presence of this signature phospholipid located in the inner mitochondrial membrane. Cardiolipin would contribute to provide a flexible amphipathic association between the two monomers of the CAC and to extend the stabilisation of the dimer, as previously proposed for the ADP/ATP carrier and UCP1 (uncoupling protein 1) [103,104]. Recent papers demonstrated that interfacial lipids help to stabilise other membrane transport proteins also, such as SLC26 and SLC23 dimers and other oligomeric membrane proteins [105,106,107]. Additionally, it has been ascertained that the impaired binding of cardiolipin can lead to the protein instability of mitochondrial carriers belonging to the SLC25 family [108]. Thus, dimerization might represent the more stable and energetically favourable option driven by differential lipid solvation [109]. Previously, it was proposed that the oligomeric state of the ADP/ATP carrier (SLC25A4) was mediated by endogenous tightly bound cardiolipins inserted between the two monomers [103]. These data can be now interpreted in the context of our observations about the missed detection of the CAC dimer by Western blotting (Figure 3). Notably, cardiolipin is also required for the correct function of the CAC [51,100,110], similar to what was proposed for the ADP/ATP carrier [97]. Therefore, all these insights and our biochemical data let us speculate that CAC can form dimeric/oligomeric structures for addressing specific physiological requirements, according to what has been observed for other transporters and/or proteins of the inner mitochondrial membranes, able to participate in supercomplexes [28,83,84,85,86].

### 4.4. Employment of Cross-Linking Agents for Investigating CAC Dimerisation

Consistent with the above-mentioned conclusions, we also investigated CAC dimerization by using cross-linker reagents. Formaldehyde (cross-linking distance = 2.3–2.7 Å), which took a sufficiently long time to induce/demonstrate the possible binding between CPT-2 and CAC in the mitoplasts [62], was ineffective in the case of the purified CAC protein, most likely due to a higher mobility and greater distance of two CAC monomers in the lipid bilayer. To demonstrate this hypothesis, it was observed that the heterobifunctional cross-linkers sulfo-MBS and sulfo-SMPB, whose spacer arms are 9.9 Å and 14.5 Å long, respectively, were effective in cross-linking two (or three) CAC monomers. The employed cross-linkers can bind lysine/arginine residues through their N-hydroxysuccinimide moiety (NHS), and cysteine residues through their maleimide group. The reconstituted protein treated with these cross-linking reagents, detected after SDS-PAGE by immunoblotting, revealed a band corresponding to a homodimer (Figure 3, lanes 3 and 6, respectively). These data suggest that two CAC monomers can associate to form dimers along conformational changes, thanks to the formation of transient networks of H-bonds, whose role is to stabilise and provide flexibility to the homodimer [53,105,106,111].

One point of interest was the immunodetection of a band at around 90 kDa after the treatment of the protein with the longer sulfo-SMPB cross-linker (Figure 3 lane 6). The trimeric structure of the CAC is, in this experiment, clearly induced by the cross-linker. However, this result let us wonder and speculate about the existence of physiological (stressing) conditions allowing CAC to assemble in higher order oligomers, as has been observed for other membrane proteins and, in particular, in secondary transport proteins [93,112,113].

The formation of the CAC’s dimeric structure in the presence of cross-linkers was also obtained with recombinant proteins, by testing different ratios of WT and C-lessV in the presence of Sulfo-SMPB. As reported in the immunoblotting of Figure 4A, an increasing amount of C-lessV led to a progressive stochastic formation of the WT homodimer, which disappeared at the ratio 25/75 WT/C-lessV. It should be stressed that the observed smears on the gel (Figure 4A) at the higher molecular weights may be due to the possible aggregation of the recombinant proteins in the presence of the hetero-cross-linking agent. Conversely, smears are totally absent in the purified mitochondrial CAC reconstituted in liposomes (i.e., in Figure 3, lane 2), in absence of sulfo-SMPB. Additionally, variations of transport activity as a function of the ratio among WT and C-lessV, at a variance with the recombinant proteins alone (Figure 4B), allows us to assume that CAC could assemble as an active dimer in the lipid bilayer. The structural data achieved with different combinations of WT and C-lessV and the functional analysis of the hetero-dimer WT/C-lessV (ratio 50/50) let us speculate that the activity of the WT/C-lessV mix (ratio 50/50) derives from the different contribution of proteins detected in correspondence with the WT-CAC dimer band, and from the C-lessV or WT monomers band, supporting the existence of a functional CAC dimer, as observed for other dimeric membrane proteins, such as SLC4, SLC23, and SLC26 [105,114]. It should also be stressed that, despite the existence of potential dimeric forms of the CAC, structural/functional analyses [36,54,115,116] have already clarified that the translocation of the substrate through the carrier occurs within the single monomer. A similar behaviour has also been described for the dimeric membrane proteins belonging to the SLC4, SLC23, and SLC26 families, as cited above [114].

### 4.5. Size-Exclusion Chromatography for Determining the Molecular Weight of the Naïve Protein Purified from Rat Liver Mitochondria

A further approach related to the molecular size of the carnitine/acylcarnitine carrier was the determination of the molecular weight of the native protein purified from rat liver mitochondria via size-exclusion chromatography (see Results). The protein, detected after SDS-PAGE by immunoblotting, was eluted from the Sephadex G-200 column as a dimeric form (Figure S1A,B). In addition, these fractions showed a conserved functional integrity when they are reconstituted in the liposomes for measuring the transport activity (Figure S1C). These further results confirm that two CAC monomers can be selectively associated, although it is not clear yet whether the association can affect or increase carrier function.

### 4.6. Crystallographic Dimers of the ADP/ATP Carrier Used as a Template to Model the CAC Dimer

Recent observations revealed that the ADP/ATP carrier forms crystallographic dimers [42] and shows a kinetic ping–pong mechanism [13]. Building on these findings, we hypothesised that the carnitine/acylcarnitine carrier (CAC) might also assemble as a homodimer or oligomer despite its ping–pong catalytic cycle, depending on specific physiological requirements.

To gain insights into the dimeric structure of the CAC, we used previously generated 3D comparative models of the CAC in both c- and m-conformations [18] to build 3D models of the CAC dimers. These models consist of dimers of two monomers in c-conformation, two in m-conformation, and combinations of c-/m-conformations, based on the crystallographic dimeric structure (4c9h.pdb) of the yeast ADP/ATP carrier [42], used as a protein template in the 3D comparative modelling analysis. Notably, the protein databank offers different crystallographic dimer orientations, with some monomers arranged in parallel and others in an antiparallel orientation. For our purposes, 4c9h.pdb provided the most coherent orientation for the CAC dimers. The rationale for superimposing our monomers onto crystallographic dimers stems from the notion that these dimers form along preferred interaction surfaces. In several cases, crystallographic dimers and oligomers have served as a starting point for identifying physiological dimers and oligomers [117,118,119].

Based on these considerations, we superimposed the 3D models of the CAC monomers in c- and m-conformations onto the single monomers of the ADP/ATP carrier crystallographic dimer to create the CAC dimer models. Cardiolipin molecules surrounding each monomer were also included in the models, derived from comparative analyses with crystallised structures of the ADP/ATP carrier in both c- and m-conformations. Specifically, the coordinates of cardiolipin molecules for CAC 3D models in c-conformation were taken from the yeast ADP/ATP carrier crystallographic dimer (4c9h.pdb),while the coordinates for cardiolipin molecules in m-conformation were sourced from the *T. thermophilus* ADP/ATP carrier (6gci.pdb [64]).

### 4.7. Energy Minimisation of the CAC Dimer 3D Models and Calculation of the Free Energy of Interaction at the CAC Monomer–Monomer Interface

The four resulting systems were embedded in a lipid bilayer composed of phosphatidylcholine (POPC), phosphatidylethanolamine (POPE), and cardiolipin, which are the main components of the inner mitochondrial membrane as described by Funai et al. [68]. These systems were then relaxed using the CHARMM36m force field [69]. The free energy of interaction at the monomer–monomer interface, after energy minimisation, indicated that the CAC dimer consisting of two monomers in m-conformation is slightly more stable than the dimer with two monomers in c-conformation. The lowest free energy of interaction was observed for the CAC dimer with one monomer in c-conformation and the other in m-conformation.

Based on the calculated interaction energy values, it can be speculated that dimerization represents an energetically favourable condition for CAC, potentially allowing more monomers to be allocated closely within the inner mitochondrial membrane. The lower energy observed for the CAC dimer with two monomers in m-conformation, compared to the dimer with two monomers in c-conformation, suggests that the favoured m-conformation dimer could counterbalance the higher stability expected for a single monomer in c-conformation, due to a stronger matrix gate compared to the c-gate. This could enhance the efficiency of substrate export from the mitochondrial matrix according to specific physiological needs [81,120].

Additionally, the lowest free energy of interaction observed for the CAC dimer with one monomer in m-conformation and the other in c-conformation (Table 2) suggests a possible relationship between the formation of this dimer, its ability to translocate substrates via a ping–pong kinetic mechanism, and a more efficient exchange of substrates across the inner mitochondrial membrane. Notably, our approach relies on estimating the free energy of interaction at the protein–protein interface after a few energy minimisation steps of the constructed complexes. This method, as recently described, provides reliable protein–protein binding affinity estimations without the need for long molecular dynamics (MD) simulations [121]. However, MD-based simulations, as well as new structures obtained by electron microscopy or X-ray diffraction, can in the future provide more details about the interactions occurring at the CAC monomer–monomer interface.

### 4.8. Physiological/Structural Requirements Justifying the Existence of the CAC Dimers/Oligomers

From our molecular modelling analysis, from the calculated free energy of interaction, and from the gels/blots presented above, it can be inferred that dimers or oligomers consisting of at least two or three CAC monomers can assemble. Based on in silico observations and in vitro transport assays on reconstituted CAC WT and the combination of the CAC WT and CAC C-lessV mutants, it appears that these assembled dimers are energetically favoured and can likely enhance substrate exchange efficiency, similar to other transporters and complexes forming supercomplexes in the inner mitochondrial membrane. In general, transient protein–protein interactions occur continuously in vivo, associating and dissociating in response to various intracellular pathophysiological conditions [122].

We expect that mitochondrial carriers can assemble as dimers or oligomers under specific conditions, as observed for AGC (without changing AGC’s function) or as anticipated for AAC, phosphate carriers, and antiporters of the mal/asp shuttle [32,33,34,35,91]. Although the substrate translocation pathway remains confined within each monomer, CAC and potentially most mitochondrial carriers could benefit from dimerization, optimising their distribution within the inner mitochondrial membrane.

Notably, the indicated network of hydrogen bond interactions provides a sufficiently weak/dynamic system, consistent with the dynamic monomer–monomer interactions proposed for the phosphate carrier by Wohlrab [123]. This H-bond interaction network can be envisioned as a zipper that can quickly and efficiently open and close in both directions, with a mobility comparable to the breaking and forming of intra/inter-repeat interactions within each monomer, facilitating substrate translocation during conformational changes [14].

### 4.9. General Considerations and Open Questions

Although mitochondrial carriers, including CAC, function as monomers with translocation pathways confined to each monomer [17,37,64], this does not preclude their ability to assemble as dimers. Our transport assays on reconstituted CAC WT/C-lessV mutant combinations support this hypothesis and offer a different interpretation from similar assays conducted by other researchers [103,124,125,126], even on other transporters [113,127].

During the equilibration steps in the lipid bilayer, it was observed that the two CAC monomers forming the dimer in the generated 3D models tend to come closer together in all simulations, rather than moving apart. This suggests that monomer–monomer attraction is greater than inter-monomer repulsion, consistent with observations for other membrane proteins. The reliability of these results is reinforced by the initial protein dimer poses obtained by constructing the 3D model of the CAC dimers based on the ADP/ATP carrier crystallographic dimer used as a template.

Among residues at the monomer–monomer interface within a 4 Å distance range, at least three basic residues are notable: R60 on the short helix h12, K267 on the matrix loop between the short helix h56 and the beginning of transmembrane helix H6, and R211 on the cytosolic loop between transmembrane helices 4 and 5 [18,81]). Notably, K267 and C58 in the lower half of the CAC dimer, as well as R211 and C283 in the upper half, are potential candidates for cross-linking to form observed cross-linked dimers, with distances ranging from 8 to 14 Å in the equilibrated structures.

Further structural analyses by X-ray or electron microscopy, or by mass spectrometry of mitochondrial membranes, and new functional evidence are needed to provide new pieces of evidence about the possible folding of mitochondrial carriers (including CAC) in dimeric forms.

We speculate that dimerization and the ping–pong mechanism enable a more efficient substrate exchange, as two substrates can enter the two CAC units in the proposed dimer, which are held together by a dynamic hydrogen bond network acting as a zipper that opens and closes in both directions while substrates cross each monomer. Additionally, the reorientation step of a putative unloaded carrier in c-state conformation during the uniport mode [11] may also be favoured by dimerization, as the activation energy barrier for conformational change would be lower than expected for a mitochondrial carrier considered as an obligatory antiporter unable to assemble as a dimer [12,54,86].

## 5. Conclusions

This study sheds light on a possible transient dimerization of the Carnitine/Acylcarnitine Carrier (CAC), a key mitochondrial transporter involved in fatty acid metabolism. Through a combination of biochemical approaches, including cross-linking and PAGE analysis, as well as in silico molecular modelling, we demonstrated that CAC could form functional homodimers without altering its ping-pong transport mechanism. Our findings suggest that while the intra-monomeric substrate translocation does not prevent carrier dimerization. It has been also proposed that the dimerization of mitochondrial carriers might play a regulatory role, potentially influencing the carrier’s stability or interaction with other mitochondrial proteins.

Moreover, the employment of milder detergents like Sarkosyl appears to preserve CAC native-like conformation during the performed analysis. The interactions of CAC with cardiolipin also appeared to play a critical role in stabilizing the dimeric form, emphasizing the importance of the lipid environment of the inner mitochondrial membrane in mitochondrial carrier function.

Future investigations are required to further explore the physiological relevance of CAC dimerization, particularly under conditions of metabolic stress or altered mitochondrial dynamics. Additionally, our work opens the door for examining whether dimerization/oligomerization have potential implications for disease states associated with CAC mutations, offering new perspectives for therapeutic intervention.

## Figures and Tables

**Table 3:** Free energy of interaction at the CAC-CAC protein–protein interface. Please note that more negative ‘interaction energy’ values, reported here in kcal/mol, indicate stronger binding interactions and thus higher binding affinity at the protein–protein interface of the indicated CAC-CAC dimeric complexes. The energy terms and contributions are reported in kcal/mol according to the FOLDX indications. Each energy term has a specific weight in the calculation of the interaction energy, according to protocols previously reported in [65,66,73,74]. The calculated energy terms and item names are reported in the first cell of each row (i.e., in the first column of the reported table), whereas items (protein names and chain ID) and the calculated energy terms are reported within columns 2–5. The number of units and decimal digits was the same as that produced by the software. For a complete explanation of item names and energy terms, please visit the following link: http://foldxsuite.crg.eu/command/AnalyseComplex, URL accessed on 27 July 2024). Abbreviations: CAC, carnitine/acylcarnitine carrier. For the number of units and decimal digits and for the statistical significance of FoldX predicted protein binding interactions, read [82].

	Dimeric CAC in c-conf Post Minimisation	Dimeric CAC in m-conf Post Minimisation	Dimeric CAC c.conf./m.conf.dx	Dimeric CAC m.conf./c.conf.dx
Group A	X (CAC c.conf)	J (CAC m.conf)	X (CAC c.conf)	J (CAC m.conf)
Group B	Y (CAC c.conf)	K (CAC m.conf)	K (CAC m.conf)	Y (CAC c.conf)
IntraclashesGroup1	26.8128	20.1334	18.5575	20.7697
IntraclashesGroup2	22.4227	26.3712	22.0363	31.8141
**Interaction Energy**	**−5.65407**	**−6.70847**	**−3.5527**	**−8.81348**
Backbone Hbond	−2.06262	−1.21455	−1.00236	−3.08866
Sidechain Hbond	−9.33346	−5.00837	−1.84149	−5.87783
Van der Waals	−6.28856	−8.61397	−4.49641	−7.60667
Electrostatics	0.611469	−0.828307	−0.358759	0.0574812
Solvation Polar	8.2012	9.68964	5.52538	9.71022
Solvation Hydrophobic	−6.85538	−12.8032	−6.35007	−9.63725
Van der Waals clashes	0.315786	0.139778	0.0171242	0.28503
Entropy sidechain	8.61905	9.46367	3.50595	6.42998
Entropy mainchain	1.71522	2.48441	1.27416	1.33332
Cis_bond	4.44 × 10^−16^	1.48 × 10^−1^	0.211593	1.11 × 10^−16^
Torsional clash	0.00915503	0.0934752	0.00694907	0.139602
Backbone clash	0.666715	1.79593	1.1584	3.18061
Helix dipole	−0.613325	0.335984	−0.0318095	−0.42639
Electrostatic kon	0.0273986	−0.594598	−0.0129609	−0.132315
Entropy Complex	2.384	2.384	2.384	2.384
Number of Residues	580	580	580	580
Interface Residues	24	37	29	29

## Data Availability

The data presented in this study are available on request from the corresponding authors.

## Supplementary Materials

The following supporting information can be downloaded at: https://www.mdpi.com/article/10.3390/biom14091158/s1, Figure S1: Determination of the molecular weight of the native CAC protein via size-exclusion chromatography.

## Abbreviations

MC (Mitochondrial Carrier), CAC (carnitine/acylcarnitine carrier), 1,4-Dithioerythritol (DTE), Sarkosyl/PAGE (N-Lauroylsarcosine polyacrylamide gel electrophoresis), SDS-PAGE (sodium dodecylsulphate polyacrylamide gel electrophoresis), Sulfo-MBS (m-maleimidobenzoyl-N-hydroxysulfosuccinimide ester), and Sulfo-SMPB (Sulfosuccinimidyl 4-(p-maleimidophenyl)butyrate).
