# Supplementary material for: Insights into Transient Dimerisation of Carnitine/Acylcarnitine Carrier (SLC25A20) from Sarkosyl/PAGE, Cross-Linking Reagents, and Comparative Modelling Analysis
Source: Biomolecules. 2024 Sep 14;14(9):1158. doi: 10.3390/biom14091158 (PMC11430254; doi:10.3390/biom14091158)

Insights into Transient Dimerisation of Carnitine/Acylcarnitine Carrier (SLC25A20) from Sarkosyl/PAGE, Cross-Linking Reagents, and Comparative Modelling Analysis

Nicola Giangregorio [1,*,†], Annamaria Tonazzi [1,†], Ciro Leonardo Pierri [2,*] and Cesare Indiveri [1,3]

1 CNR Institute of Biomembranes, Bioenergetics and Molecular Biotechnologies (IBIOM), Via Amendola 122/O, 70126 Bari, Italy; a.tonazzi@ibiom.cnr.it (A.T.); c.indiveri@ibiom.cnr.it (C.I.)
2 Department of Pharmacy—Pharmaceutical Sciences, University of Bari, Via E. Orabona, 4, 70125 Bari, Italy
3 Department DiBEST (Biologia, Ecologia, Scienze della Terra) Unit of Biochemistry and Molecular Biotechnology, University of Calabria, Via Bucci 4C, 87036 Arcavacata di Rende, Italy
* Correspondence: n.giangregorio@ibiom.cnr.it (N.G.); ciro.pierri@uniba.it (C.L.P.); Tel.: +39-080-5442789 (N.G.) ; Tel: +39-080-5443614 (C.L.P.)
† These authors contributed equally to this work.

Figure S1.

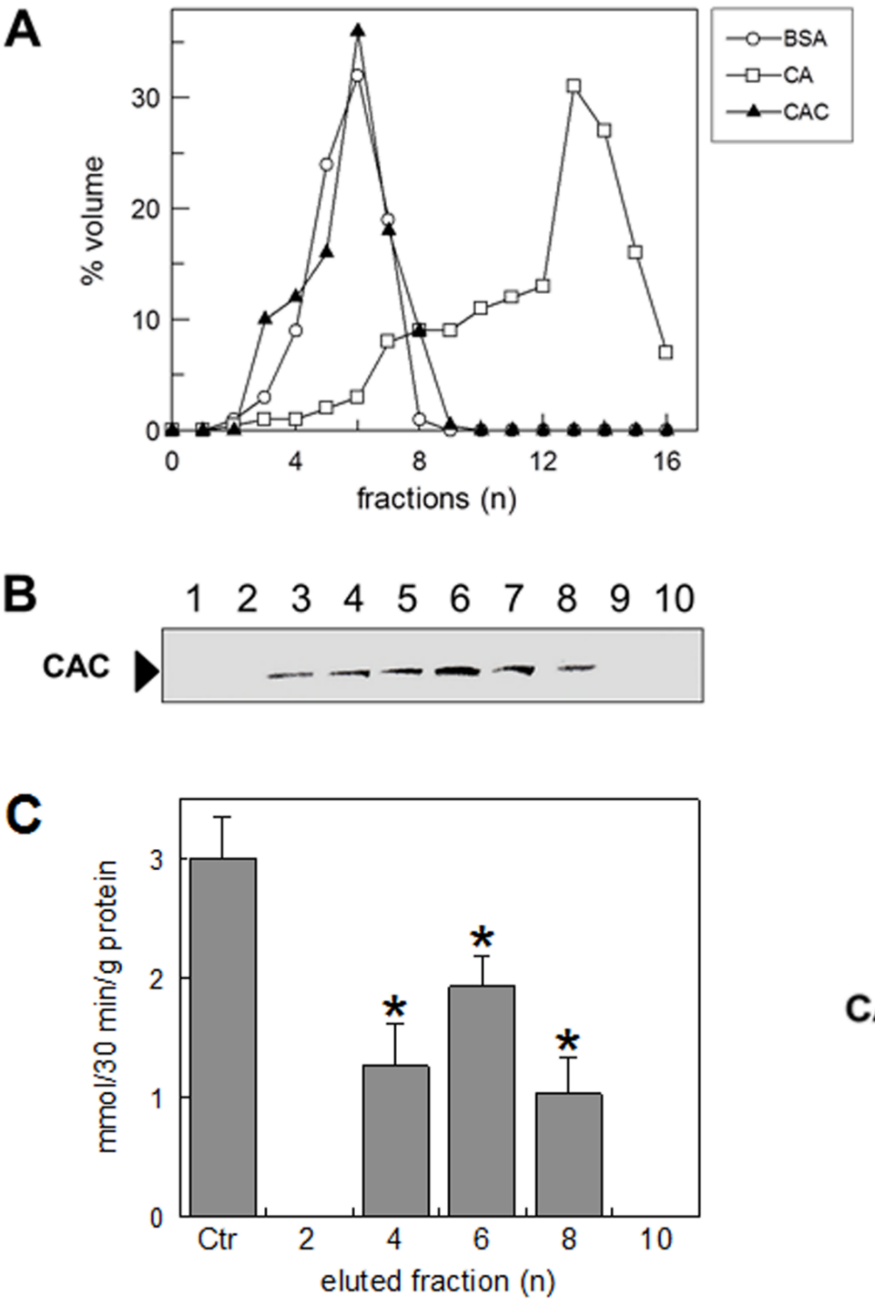

Figure S1. Determination of the molecular weight of the native CAC protein via size-exclusion chromatography. A) Native protein purified from rat liver mitochondria (300 μl) was applied to a Sephadex G-200 gel filtration column, and the elution profile detected by immunoblotting B) was compared with the elution profiles of BSA and Carbonic Anhydrase (for details, see Materials and Methods), see also Figure 4A. C) The transport activity of 300 μl purified reconstituted mitochondrial CAC before separation by Sephadex G-200 gel filtration (Ctr) and 300 μl of some protein fractions collected during elution of the column (2, 4, 6, 8 and 10 fractions), expressed as mmol/30 min/g protein, was measured. The values are the means ± SD from three independent experiments; significantly different from the respective control as estimated by Student's t test (* $p < 0.01$). A full representation of Figure 1SB is reported here below.

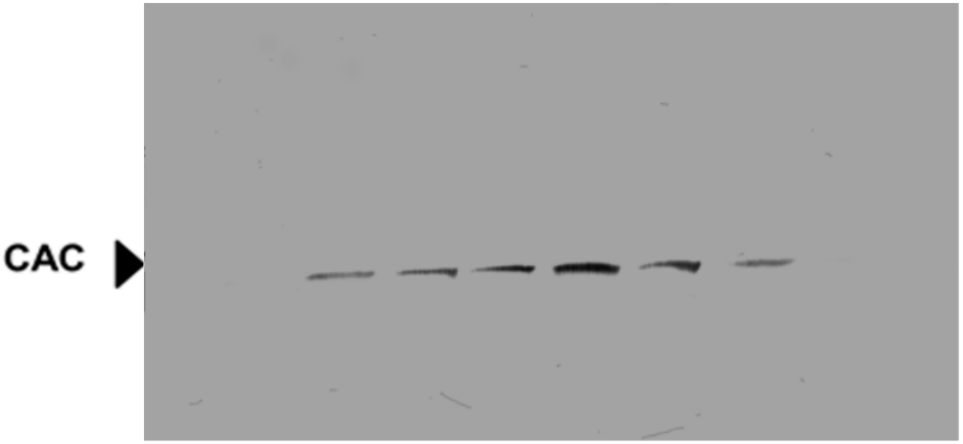

Fig. S2A

Fig. S2B

Uncropped blots

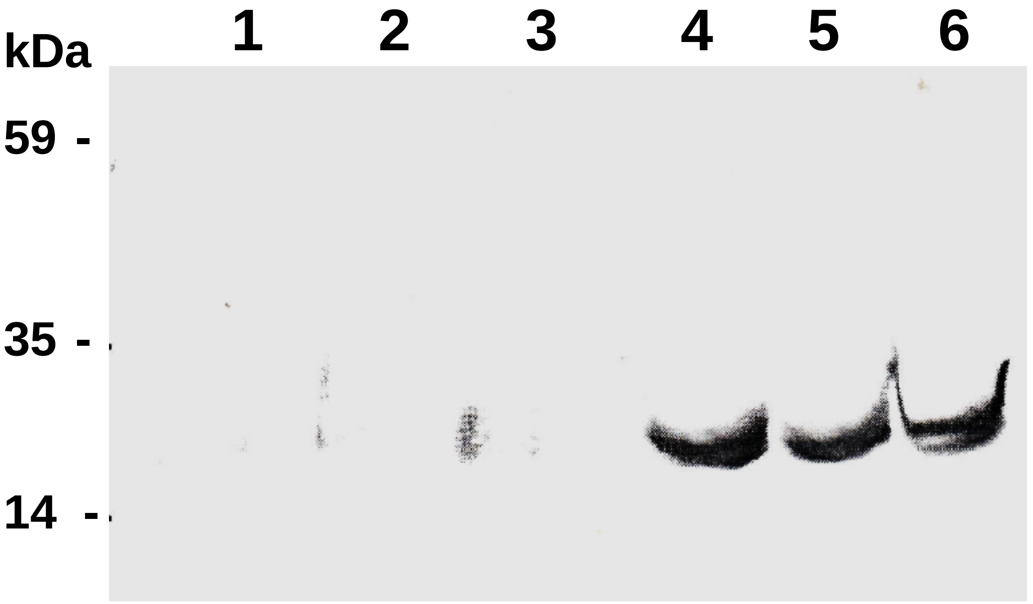

Lanes 1 and 2 are those represented in Fig. 2A
Lanes 3 and 4 are those represented in Fig. 2B
Lanes 5 and 6 are replicates of lane 4

Fig. S2C

Uncropped blot

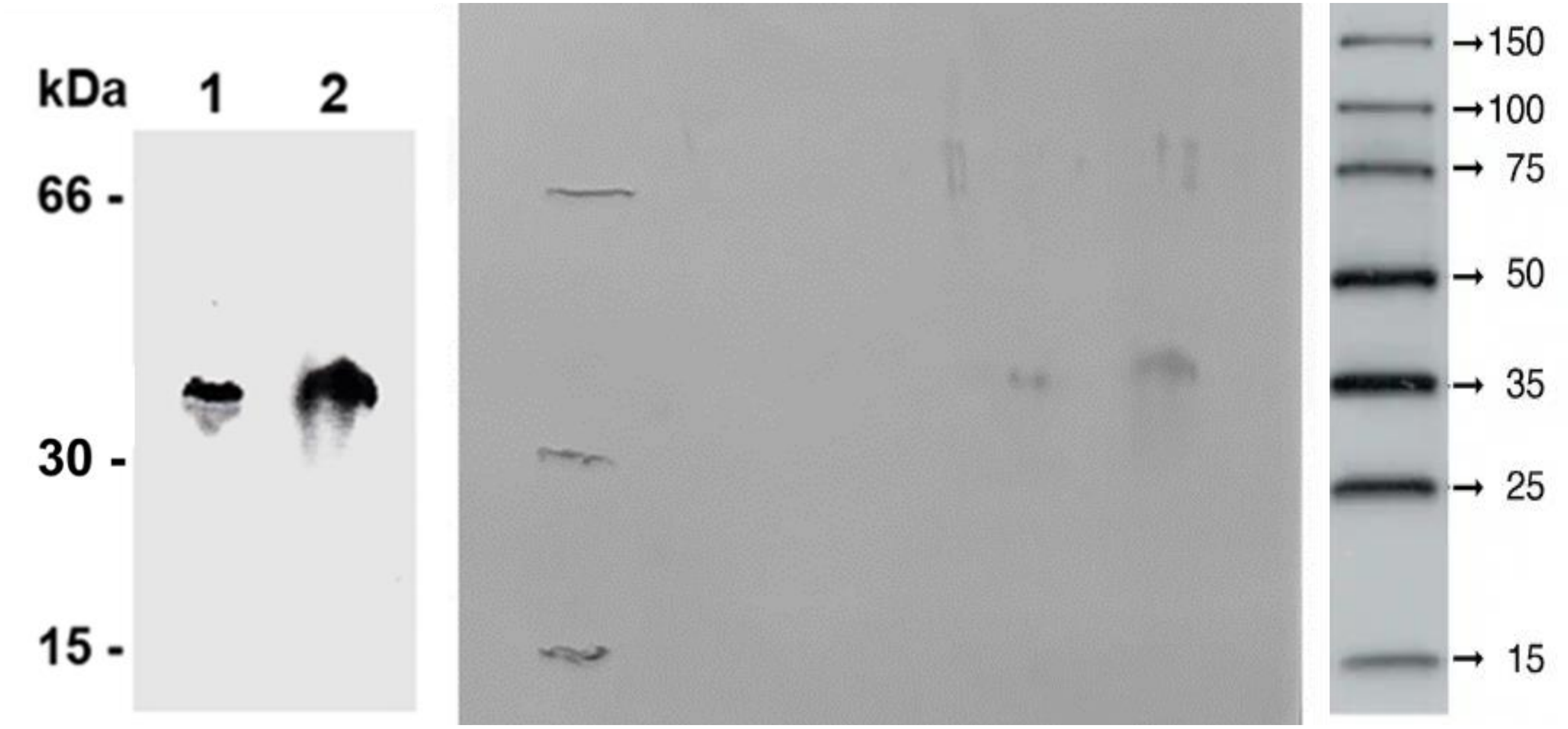

The 30 kDA marker in the middle panel was manually reported (with a pen) and that is the reason for which it looks like a bit lower than the number indicated in Fig. 2C. For comparative purposes, on the right has been reported a commercial marker for WB

Fig. S4

Lanes 1,2,3 or the entire blot represent replicates of the lanes 1,2, 3 reported in Fig. 4 , whose lane 4 corresponds to the 7th lane of the entire blot

Uncropped blot

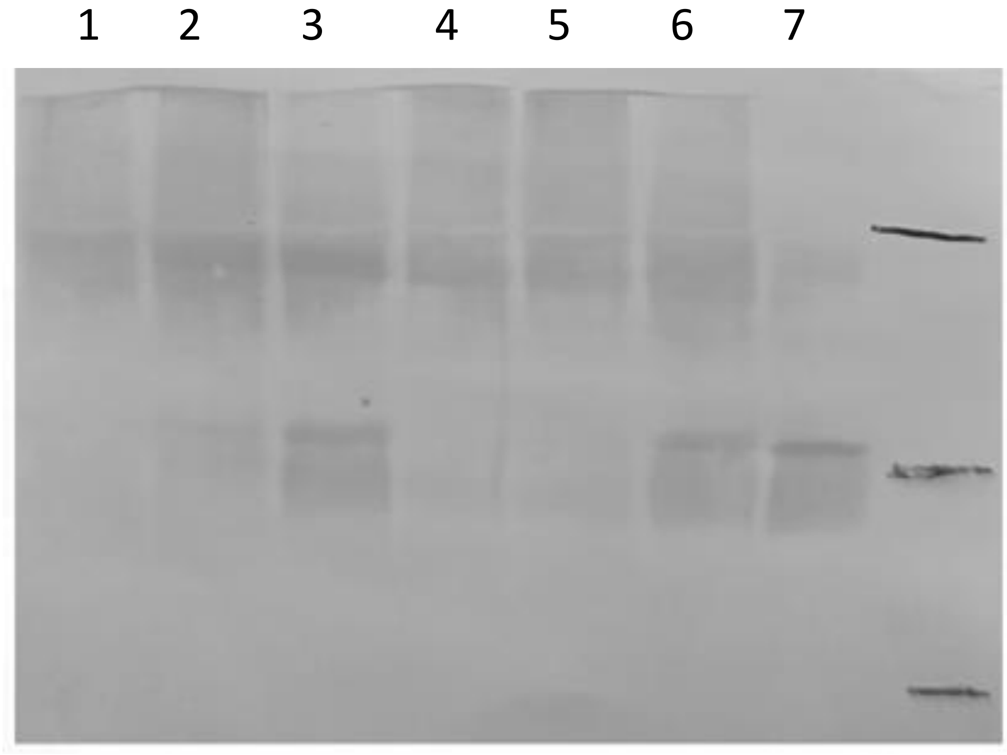

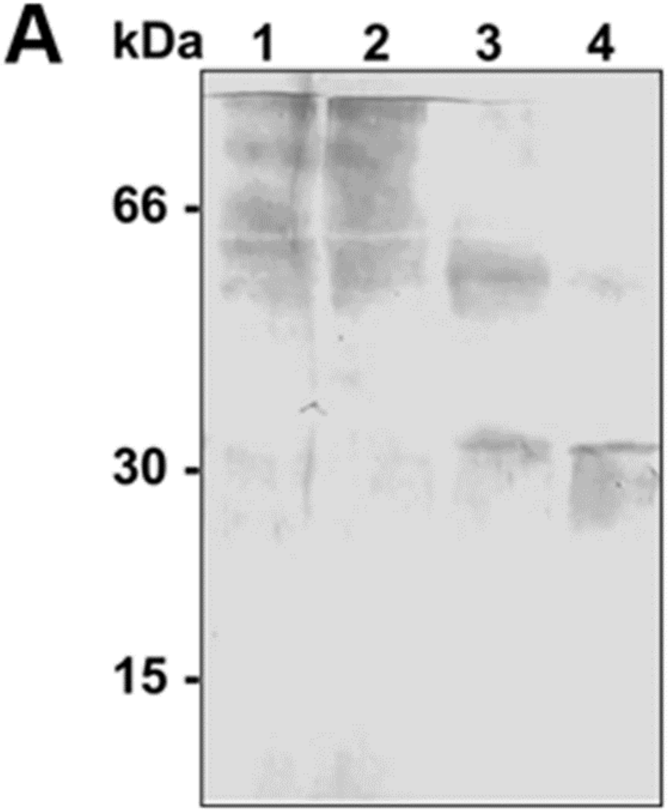

This is the entire blot of Fig. S1, also reported at the end of Supp. Mat. doc

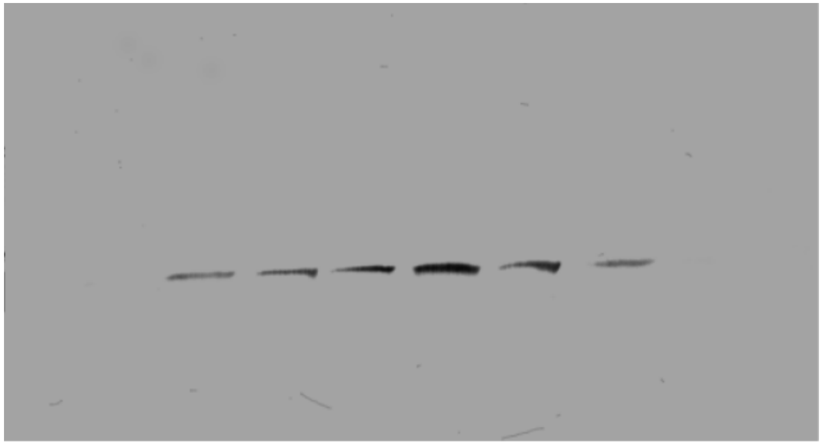

Supplement: Supplementary file 1 [file biomolecules-14-01158-s001.zip › biomolecules-3134145-supplementary.pdf]
